# Health care providers’ understanding of self-management support for people with chronic low back pain in Ethiopia: an interpretive description

**DOI:** 10.1186/s12913-022-07610-5

**Published:** 2022-02-14

**Authors:** Mulugeta Bayisa Chala, Jordan Miller, Setareh Ghahari, Yemataw Wondie, Abey Abebe, Catherine Donnelly

**Affiliations:** 1 Queen’s University, School of Rehabilitation Therapy, Kingston, ON Canada; 2 Department of Physiotherapy, College of Medicine and Health Sciences, University of Gondar, Gondar, Ethiopia; 3 Department of Psychology, College of Social Sciences and Humanities, University of Gondar, Gondar, Ethiopia

**Keywords:** Self-management, Self-management support, Chronic low back pain, Interpretive description, Ethiopia

## Abstract

**Background:**

Healthcare providers play a key role in supporting people with chronic low back pain to self-manage their condition. The study aimed at exploring how health care providers understand and conceptualize self-management and how they provide self-management support for people with chronic low back pain in Ethiopia.

**Methods:**

Health care providers who have supported people with low back pain, including medical doctors and physiotherapists, were approached and recruited from three hospitals in Ethiopia. This study employed an interpretive descriptive approach using semi-structured interviews.

**Findings:**

Twenty-four participants (7 women; 17 men) with a median age of 28 (range 24 to 42) years and a median of 9.5 years (range 1 to 11 years) of helping people with chronic low back pain were interviewed. Seven major themes related to health care providers’ understanding of self-management support for people with chronic low back pain in Ethiopia emerged. The findings show that self-management was a new concept to many and health care providers’ had a fragmented understanding of self-management. They used or suggested several self-management support strategies to help people with CLBP self-manage their condition without necessarily focusing on enhancing their self-efficacy skills. The participants also discussed several challenges to facilitate self-management support for people with chronic low back pain. Despite the lack of training on the concept, the providers discussed the potential of providing self-management support for people with the condition.

**Conclusions:**

Self-management was a new concept to health care providers. The providers lack the competencies to provide self-management support for people with chronic low back pain. There is a need to enhance the health care providers’ self-management support competencies through training.

**Supplementary Information:**

The online version contains supplementary material available at 10.1186/s12913-022-07610-5.

## Introduction

Low back pain (LBP) is the single largest contributor to years lived with disability globally [1, 2]. People with persistent and recurrent LBP experience long-term disability, lost productivity, and associated medical costs [3, 4]. The burden of disabling LBP is increasing globally despite the recent advances in imaging and the availability of biomedical, pharmacological, surgical, and rehabilitation management options [5–8].

While there is generally an upward trend in the global prevalence of LBP-related disability [5], these numbers are projected to increase exponentially in low-and-middle-income countries due to the increasing use of ineffective pain management strategies [9]. Moreover, the literature indicates that the health systems in these countries face the dual burden of infectious and chronic non-communicable diseases [10]. Given the actual and imminent health risks, infectious diseases are prioritized over musculoskeletal conditions such as LBP [11]. In Ethiopia, LBP is one of the major public health problems [12–14]. Still, the country lacks policies and strategies to address the health needs of people experiencing this condition [12].

Research on prognosis for chronic musculoskeletal pain conditions (> 3 months duration), such as chronic low back pain (CLBP), suggests complete resolution is unlikely [15–17]. Therefore, the focus should be on supporting people to manage their condition rather than finding a cure [18, 19]. Multiple studies indicate self-management support is an effective approach to manage CLBP and related disability [19–21], and clinical practice guidelines recommend self-management support for people living with CLBP [22–24].

Adams et al. (2004) described self-management support as “the systematic provision of education and supportive interventions by health care staff to increase patients’ skills and confidence in managing their health problems, including regular assessment or progress and problems, goal setting, and problem-solving support” [25]. A central tenet of self-management support is to help patients engage in their care decision and develop the skills to better manage their condition independently or through a partnership with their health care providers (HCPs) [26, 27]. As such, self-management support aims at enhancing patient’s self-management skills for the day-to-day management of their health problems (e.g., pain) and improve their quality of life [28, 29]. These skills include problem-solving, decision-making, resource utilization, the formation of a patient-provider partnership, action planning, and self-tailoring [26].

Although self-management is considered an effective approach to care for people with chronic health conditions, the term is ambiguous and lacks a universally accepted definition [30–32]. As described by MacGown (2005), self-management “often means different things to different people- and sometimes different things at different times even to the same people” (MacGown, 2005, page.1) [32]. Furthermore, there is a lack of consensus on what constitutes self-management support strategies for people with CLBP [33, 34], particularly in low and middle-income countries.

It is unknown if there are regional or geographical variations in the conceptualization of the term self-management. The existing definition and conceptualization of self-management varied depending on several attributes, such as the primary focus of the intervention, its process and outcomes, and the roles and responsibilities of both the patients and the providers [26, 32, 35].

Despite the increasing use of self-management support to mitigate pain-related disability among people with CLBP in other parts of the world [33, 36, 37], there is no evidence of its use in the care of people with a similar condition in Ethiopia. Although very scarce, there is evidence of self-management supports among people with chronic health conditions such as diabetes in Ethiopia [38, 39]. To date, there is no data on what strategies HCPs use to facilitate self-management support for people with CLBP in Ethiopia. Furthermore, it is unclear how HCPs define and conceptualize CLBP self-management throughout the country. A recent survey on LBP in Ethiopia suggested overutilization of guideline non-concordant care and underutilization of evidence-based care such as self-management supports for LBP [12]. The survey indicated that people with LBP received pharmacological interventions, surgery, bed rest, and back support as the first line of management for their pain [12]. Additionally, the survey suggested that people with LBP utilize alternative and traditional healing techniques such as holy water, massages, and cupping to manage their condition [12].

Therefore, the purpose of this qualitative inquiry was two-fold: 1) to explore how HCPs in Ethiopia understand and conceptualize self-management; 2) to explore how HCPs provide self-management support for people with CLBP in Ethiopia. Data from this study will inform the design and implementation of a self-management program for an Ethiopian context.

## Methods

### Study design

An interpretive description approach was used in this qualitative inquiry [40, 41]. The interpretive description draws from tenets of constructivist and naturalistic paradigm to generate applicable knowledge in health disciplines [40, 42]. The epistemological foundations of interpretive description are grounded on the researcher’s disciplinary background to yield legitimate knowledge that can be applied in a particular clinical situation [40]. As Thorne suggested, “the researcher’s theoretical affinities, disciplinary background, and orientation powerfully shapes thinking, enacting research, and the making of a research product” (Thorne 2016, P.73) [41]. Interpretive description acknowledges the theoretical and practical knowledge a researcher brings into the field [43, 44]. This approach enabled us to explore health care providers’ understanding of self-management for people with CLBP in Ethiopia, which would have been difficult to address with other traditional qualitative approaches [41, 43].

### Research team

A research team with multiple health professional background conducted this study. The lead author (MBC) completed this research as part of his doctoral thesis project. Authors, MBC and AA are physiotherapists who have clinical expertise with people experiencing LBP in Ethiopia. JM is a Canadian physiotherapist with a research focus on reducing pain-related disability through interventions such as self-management supports. CD is an occupational therapist whose clinical research focuses on team based-primary care in Canada. SG is also an occupational therapist with a research focus on the self-management of people with chronic health conditions. Lastly, YW is a clinical psychologist and researcher based in Ethiopia.

### Reflexivity statement

The participants were aware of the interviewer’s (MBC) disciplinary background, a physiotherapist who has experience providing care for people with LBP at the University of Gondar hospital in Ethiopia, and his present status as a graduate student in Canada. This has helped in building trust with the participants through shared experiences of working in the same clinical environment. Thorne et al. (2004) explains that researcher’s disciplinary orientation shapes the rigor and purpose of interpretive description [40]. In that sense, MBC, who is a physiotherapist with first-hand experience of working with people with CLBP at one of the sites, provides an insider perspective to enhance the interpretation of the data related to the research question.

### Study participants and data collection process

#### Participant recruitment

This study recruited health care providers that most commonly support people with LBP in Ethiopia. HCPs consisting of medical doctors and physiotherapists were recruited from three hospitals in Ethiopia: the University of Gondar referral hospital, Bahirdar Felegehiwot referral hospital, and Black Lion referral hospital. Recruitment took place at these hospitals as they receive the highest number of patient referrals for people with LBP from general hospitals throughout Ethiopia. Secondly, these hospitals are staffed with HCPs with various professional backgrounds and specializations who have experience providing care for people with CLBP. The participants were recruited through a purposive sampling strategy [45]. Participant’s gender, professional background (e.g., physiotherapists, physicians), and specializations (e.g., general practitioners, residents, neurologists, neurosurgeons, and orthopedic specialists) were considered to achieve maximum variation among the health professionals who manage LBP in the three participating hospitals. Participants were eligible if they were working in one of the three hospitals, involved in providing care for people with CLBP, and communicated in Amharic, the official language of the Federal Democratic Republic of Ethiopia [46].

#### Data collection

Data were collected using open-ended semi-structured face-to-face interviews [46]. The semi-structured interview guide was prepared in English and translated to Amharic after a thorough review of relevant literature on chronic low back pain self-management support. The guide was compiled by MBC and approved by all co-authors. The Amharic guide was piloted with two HCPs (a physiotherapist and general practitioner). See Additional file 1 for an English version of the interview guides. All interviews were conducted by the first author (MBC) in the hospital settings where the HCPs were working between July and November 2019. The interviews were audio-recorded, and field notes were taken to supplement each interview. The concept of information power was used to determine when to stop interviewing more participants [47]. Participants’ demographic information was collected prior to each interview.

### Data analysis

The interviews were transcribed verbatim in Amharic, and transcripts were compared to the recordings for accuracy. Transcripts and field notes were analyzed using an inductive thematic analysis approach [48]. NVivo 12 data management software (QSR International, Pty Ltd.) [49] was used to organize and analyze data. There were six steps to the analytic process: 1) familiarization with the data, 2 (generating initial codes, 3) searching for themes, 4) reviewing themes, 5) defining and naming themes, 6) writing and discussing the findings. Steps 1–4 were conducted in Amharic to stay close to the participant’s language and retain meanings [50]. The analysis started with data *familiarization* (step 1) by listening to the audio recordings, reading, and rereading the transcripts [48].

MBC and AA completed the second step of the analysis (*generating initial codes*). The two authors independently coded three interview transcripts and met to ensure consistency in the coding scheme. Frequent meetings, debriefings, and reflexive dialogues on the coding process were held with the larger research team. MBC coded all the remaining interviews for consistency while maintaining discussions with the rest of the research team.

The third step (*searching for themes*) involved bringing together codes into potential themes. The fourth step (*reviewing themes*) involved generating a thematic map by evaluating whether the themes align with the coded extracts and the entire data set [48]. At this stage, the generated themes and associated participants’ quotes were translated into English to engage the rest of the research team (JM, CD, and SG), who do not speak Amharic in the next steps of the analysis process.

In the fifth step, major themes and sub-themes were identified through an inductive approach, consistent with interpretive description (*defining and naming themes*) [40, 42, 51–53]. Themes were reviewed and finalized by the entire research team. At this stage, data evolved into a meaningful concept that explains how HCPs conceptualize self-management and identify what strategies they use to support people with CLBP to self-manage in Ethiopia. The last step of the analysis (*writing and discussing the findings*) involved choosing compelling and representative participant quotes and discussing them in relation to the study’s objective and then comparing and contrasting the results with the existing literature on self-management for chronic LBP.

#### Rigor and trustworthiness

According to Thorne et al. (2004), the credibility of the results in interpretive description is derived from the researchers’ methodological and analytic decisions [40]. In accordance with that, we used a number of strategies to maintain rigor and trustworthiness in this study. First of all, we made a considerable effort to corroborate the themes with the participant’s quotes, hence the credibility and conformability of the findings [54]. Second, we held frequent meetings and peer debriefings among the research team to enhance analytic rigor. Third, we conducted a significant portion of the analysis in the interview language to stay close to the participant’s account and minimize meaning loss before translating into English [50]. Fourth, we used the consolidated criteria for reporting qualitative research (COREQ) checklist to report the findings of this study in an effort to enhance transparency [55].

### Findings

Twenty-four HCPs participated in interviews (mean 45 min in duration). Table 1 depicts the socio-demographic characteristics of the study participants.

**Table 1:** Socio-demographic information of the healthcare providers (*n* = 24)

Characteristics	Description (frequency)
**Median age in years (Range)**	28 (24–42)
**Gender**	
Men	17
Women	7
**Discipline**	
Physiotherapy	12
Medical doctor	12
**Professional background and specialization**	
BSc PT	4
MSc PT	6
DPT	2
General Practitioner	2
Resident (R1 & R3)	2
Neurologist	2
Neurosurgeon	2
Orthopedic surgeon	4
**Median years in clinical service (range)**	9.5 (1,11)

*BSc* Bachelor of Science, *MSc* Master of Science, *PT* physiotherapy, *DPT* Doctor of physiotherapy.

**Fig. 1:**
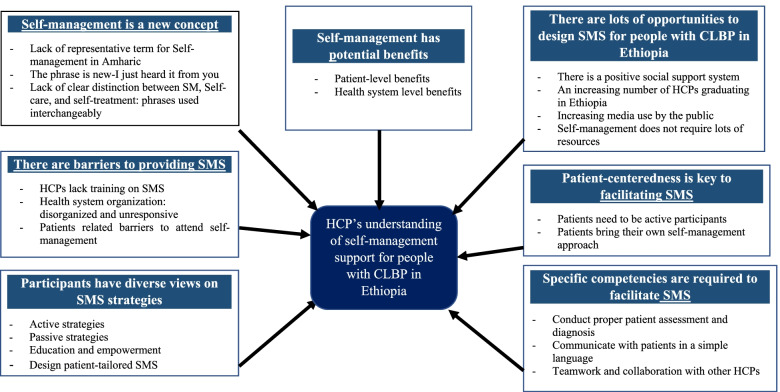
Major themes and sub-themes related to health care providers’ understanding of self-management support in Ethiopia. SM: self-management, SMS: self-management support, HCP: health care provider

Seven main themes and subthemes related to the health care providers’ understanding of self-management and self-management support for people with CLBP were identified. See Fig. 1 for the major themes and sub-themes. Additional participants’ quotes with themes and sub-themes is also presented in the Additional file 1 section of the document.

#### Self-management is a new concept

Data from our participants suggest that self-management is a relatively new concept in the Ethiopian health care environment. It is a complex term to translate into Amharic, and its definition may result in overlapping concepts with self-care and self-treatment.

The majority of the participants highlighted the lack of a representative term for self-management in Amharic. However, some HCPs have an understanding of the concept, such as the patient’s central role to manage their health condition. A physiotherapist participant described this phenomenon as; “*We may not have a single word for it in Amharic. But it is a form of self-treatment where patients take initiatives to treat themselves*.” [HCP 019; Physiotherapist].

Although the participants had some understanding of the concept underlying self-management and utilized some self-management strategies to support people with CLBP, the majority of them stated that they heard the term ‘self-management’ for the first time during the interview. This is suggestive that. One participant said, “*It is for the first time that I hear about self-management, specifically on low back pain. But as I told you, we have been doing this indirectly-as we always tell them [patients] to manage themselves. It is like that. But as a phrase, I just heard it today.*” [HCP 005; Orthopedic surgery resident (R1)].

The findings suggest that the term ‘self-management’ lacks clarity in Amharic and the phrase is used interchangeably with self-care and self-treatment. The central focus during the translation and conceptualization of self-management into the local context was based on patient’s prominent roles in managing their condition.

#### Self-management has potential benefits

Participants described several benefits of self-management support for people with CLBP, which were discussed both at patient-level and the health system level.

The participants suggested that self-management will result in better health outcomes and fewer hospital visits. One participant suggested, “*The goal is to make him psychologically, physically, and socially functional. If there is pain, we manage the pain, and if there are other things, we make them functionally independent- that is the outcome*.” [HCP 010; Physiotherapist].

The reduced number of visits would reduce the financial burden on the individual and reduce the burden on the health care providers and the health system. As one participant described, patients who self-manage their condition depend less on HCPs and make few out-of-pocket payments to access care: “… *with self-management, the patient has a clear understanding of ...hmm...their problem. They will not see a physiotherapist every time they experience pain. So, self-management reduces cost for the patient*.” [HCP 004; Physiotherapist].

At the health system level, participants indicated that self-management reduces the frequency of patient visits to the hospitals, which ultimately reduces the health system’s burden and improves care efficiency. One orthopedic surgeon said: “*You have seen it earlier...it [so many patients waiting in the outpatient department] is too much. What you do is to see them as quickly as possible and go to the next patient. So, you may not be able to see the patient properly. Self-management can reduce the load on a physician and the hospital … it is also good for the patient...that is the advantage*” [HCP 015, Orthopedic surgeon].

#### Patient-centeredness is key to facilitating self-management support

Patient-centeredness was considered a key component of self-management support for people with CLBP. Participants explained that self-management should be designed based on the patient’s needs. They also stated that self-management support successes depend on the level of the patient’s engagement in the self-management process. The following paragraphs describe the attributes of patient-centeredness in the self-management intervention for people with CLBP.

The participants acknowledged that people with CLBP have a significant role in their self-management. Most importantly, the participants discussed that such intervention requires patients to adopt an active role over a passive role to successfully manage their condition: “*well when you hear the term self-management, it means someone is not taking a sick role. It is about them [the patients] doing something …*.” [HCP 016; Neurosurgeon].

In this study, the participants talked about the importance of acknowledging and validating patient’s own pain management strategies (e.g., religious and traditional healing rituals) as key to facilitating self-management support. For instance, the providers would accommodate patient’s self-management strategies as far as it is safe and does not put them in danger; “… *I will not stop them if they tell me that they are going for holy water. Because I believe that patients can get better if they follow their faith. But I try to advise them not to engage in harmful practices*.” [HCP 004; Physiotherapist].

#### There are barriers to providing self-management support

The participants described a few challenges to designing, implementing, and evaluating self-management intervention for people with CLBP in Ethiopia. The barriers were discussed at three levels: health care providers, patients, and health systems levels.

Health care providers’ related barriers to facilitating self-management support are associated with a lack of perceived competence to design and deliver self-management support for people with CLBP. The participants stated that they never had formal training on this concept: “*How can you support patients if you do not know about it [self-management]? First of all, not every professional has good knowledge about it. I think most of them [health care professionals] are new to this concept.”* [HCP 003; Physiotherapist].

Additionally, a few participants also indicated that self-management is not well embraced by their colleagues, suggesting attitudinal barriers to self-management among HCPs in Ethiopia. One neurologist expressed this as follows: “...*they laugh at me when I bring this topic [self-management] on a seminar series. They say, ‘our people will not accept this.’ But that is not how I would like to think. I have applied this to myself [for his chronic pain]. It is not that difficult to do it unless we believe it is.”* [HCP 018; Neurologist].

Our participants stated that the current health system is disorganized and unresponsive to provide appropriate self-management support for people with CLBP due to heavy workload, lack of self-management culture within the health system, and the absence of a mechanism to evaluate whether such intervention works not. One participant said, “*Usually, it is because the outpatient department is jammed. There are lots of patients in the clinic, both in the mornings and afternoons. You just do not have time to sit and talk to them*.” [HCP 007; Neurologist].

Barriers to referring between departments and providers was seen to make it more difficult to referring patients to the providers who are most likely to provide self-management supports. One participant explained this barrier as follows: “… *a neurologist may see the patient and may not link them to physiotherapy. Or sometimes, the patients come with little or no information on their chart. The referral channel is not well established-it is not centralized. As I told you before, there is no system that connects all the outpatient departments to each clinic within the hospital.”* [HCP 010; Physiotherapist].

Finally, participants in this study identified several patient-related barriers to attend self-management. One of the factors that could influence patient’s acceptance and engagement in self-management is their attitude toward such intervention and their expectation of health care. An orthopedic surgeon explained this attitudinal barrier as follows: “*The majority of patients do not believe in such treatment [self-management] unless you prescribe them some medications or do something with your hand. There is a perception problem. When you tell them to self-manage it at home, they say ‘I am here because I am sick*.’” [HCP 012; Orthopedic surgeon].

Furthermore, participants speculated that patients with a low level of education and those from rural areas find self-management support challenging to follow compared to those from urban areas: “*Patients from urban settings have relatively better information. They read and come, or they will listen to what you have to say. They will compare the information and will challenge you. People from the rural area, for example, farmers do not have time and space to do the exercises*.” [HCP 004; Physiotherapist].

#### Participants have diverse views on self-management support

Participants in this study reported using different strategies to support people in managing their CLBP. The strategies included active and passive approaches, patient education, and designing patient-tailored self-management interventions.

Encouraging patients to engage in physical activities or activities of daily living and postural correction were discussed as active self-management support strategies. The following quote by a physiotherapist participant highlights the use of exercise in the self-management of people with CLBP: “*They can use exercises as self-management. For example, you can advise patients to do aerobic exercises to reduce their body weight or strengthen their abdominal muscles. You can tell them to go for a walk – that is also self-management*.” [HCP 021; Physiotherapist].

Some of the frequently mentioned passive self-management support strategies included pain medication, modalities (e.g., heat, tens), and back supports (both traditional and modern corsets or braces). One resident doctor said, “*We advise them to take pain medications for their back- that can be part of self-management. As I told you, we have limitations in other approaches [other self-management strategies]- it is undeniable. So, we write them a prescription and advise them to buy pain medications when they need it.”* [HCP 005; Orthopedic surgery resident (R1)].

The participants talked about the importance of reassurance and pain education as core support strategies for people with CLBP. The following quote by an orthopedic surgery resident illuminates how they use patient education as a self-management support for people with CLBP. They said, “*As I said before, we educate them ‘these are the reason for their pain- that they get better or worse if they do this or that ...’ We empower them by telling them they are the ones who know the changes [prognosis]-that is how we involve them in their self-management*.” [HCP 005; Orthopedic surgery resident (R1)].

Finally, most of our respondents discussed the importance of designing a tailored and patient-specific self-management support by considering every patient’s uniqueness with CLBP. The following quote by a physiotherapist explains this concept: “*We must know that every patient is different. Not all people with LBP come with the same symptom or seek the same treatment or self-management. I think we must try to design individualized treatment for every patient*.” [HCP 009; Physiotherapist].

#### Specific competencies are required to facilitate self-management support

The participants suggested that self-management support should be designed and delivered by competent health care professionals who have a collective set of knowledge, skills, and attitudes related to self-management support.

Conducting proper patient assessment and pain diagnosis was discussed as a core competency required by the HCPs to facilitate self-management support. The participants acknowledged that CLBP is a complex and challenging condition for patients. To address this complexity, the participants state that HCPs require the competency to diagnose patient’s source of pain, which is evident beyond the physical symptoms. One participant said: “*Chronic low back pain is a challenging condition. In a few patients, the mental health aspect may dominate their physical one. That is why conventional physiotherapy may not be the answer -I believe most patients require a different approach. In addition to the routine physical examinations, further assessments such as their psychological, social, work, and their lifestyle evaluations have to be done.*” [HCP 023; Physiotherapist].

A few participants expressed their frustration when they could not establish a diagnosis for patients with complex CLBP. When arriving at a proper diagnosis was not possible, the HCPs referred patients to other specialists. One participant said, “*They go [patients] here and there...sometimes you may not see anything on their imaging. So, you get frustrated and refer them to another person [health care professional] or clinic who can help them [health care professional]. It is common*.” [HCP 015; Orthopedic surgeon].

Participants in this study suggested that effective communication skills are needed to facilitate self-management support for people with CLBP. They reiterated that HCPs must use a clear, simple, and contextualized language based on the patient’s socio-cultural backgrounds during self-management support provision. One participant said, “*As I said before, patient communication has a vital role in self-management intervention. For example, you should be able to communicate with a farmer from a rural area similar to an engineer working in a city. It is our role to use language that suits each patient.”* [HCP 002; Physiotherapist].

Finally, the participants considered teamwork and collaboration with other HCPs essential competencies to facilitate self-management support for people with CLBP. In particular, collaborating with other HCPs was deemed necessary to ensure that the providers involved in caring for people with CLBP work towards the same goals: *addressing patient’s concerns*.

The following quote by one of the participants elaborates on this competency: “*What I am saying is that it is better to work in a team. Because, to me, for example, my self-management advice for this patient was to quit his job. But what if he goes to a neurologist and they give him another option? They may say to him something like, ‘no you can do this and that and keep working?’ If he goes to an orthopedic doctor, they may give him a third option. That confuses the patient. So, it is better to make such decisions in a team instead of doing it alone. Self-management intervention programs work better when delivered in a team.*” [HCP 002; Physiotherapist].

#### There are a lot of opportunities to design self-management support for people with CLBP

Despite the findings that the concept of self-management was new to many participants and several perceived impeding factors to facilitate self-management support for people with CLBP in Ethiopia, the participants explained numerous opportunities to design and implement self-management support for people with CLBP.

The participants explained a strong sense of social support in Ethiopia can be a valuable asset for self-management support for people with CLBP. According to the participants, positive social support from family members, friends, community, and peers optimizes the self-management of people with CLBP. The following quote by a neurosurgeon explains this concept: “*In our community, people know how to live together and support each other. We can form a support group for people with low back pain. We can introduce the patients to each other. That is good because they can attend a group pain education and exercises.”* [HCP 016; Neurosurgeon].

It was further discussed that HCPs could be equipped with self-management competencies by making self-management content an integral part of the health care providers’ education training. One participant said, “*I believe health care professionals must have that competency [self-management support]. It has to be included in a curriculum too*.” [HCP 005; Orthopedic surgery resident (R1)].

A few participants in this study discussed the importance of harnessing digital media’s power to facilitate self-management support for people with CLBP. For instance, an increased access to the internet in Ethiopia was considered an opportunity to direct patients to online self-management-related resources. Another participant talked about the convenience of providing self-management support in a video format for people with CLBP. They said, “… *people in the cities have access to technologies. We can give them some exercises in a video format and advise them to follow it. It is very convenient*.” [HCP 020; Physiotherapist].

Finally, most of our participants suggested CLBP self-management can be delivered using fewer resources. The participants described that patients do not need expensive equipment to self-manage their back pain. In particular, in this study, physiotherapist respondents speculated that patients could do different exercises without using exercise equipment to manage their pain.

## Discussion

This is the first study investigating health care providers’ understanding of self-management for people with CLBP in Ethiopia. The key themes identified in this study adds to the existing literature by providing a context on how the providers conceptualize self-management and the kinds of strategies they use to support their patients to self-manage.

We found that the HCPs have a fragmented knowledge of self-management and self-management support for people with CLBP. HCPs use the terms self-management, self-care, and self-treatment interchangeably. This is in part due to the lack of a direct Amharic translation for self-management. The findings of this study are not unique to our research. Previous literature from other regions also indicated that the term self-management is ambiguous that lacks clarity and is open to interpretation [21, 32, 56]. Evidence shows that self-care and self-treatment have been used in association or as a surrogate term with self-management [57–59]. However, these concepts vary in their aims, nature of interventions, and the level of patient engagement in the process [35, 60]. The absence of conceptual clarity of the term self-management in Amharic can affect its usability in the Ethiopian clinical and research settings. The significance of this finding warrants the need to enhance conceptual clarity on self-management for Ethiopian HCPs through informal (e.g., ongoing continuous professional development) and formal university level (e.g., integrating self-management in the medical and allied health curriculum) training.

As discussed by our participants, self-management support has potential benefits for people with CLBP and the health system. Similar to what was reported in previous studies, the health care providers speculated that self-management support improves function and prevents further disability related to CLBP [59, 61]. Furthermore, the participants perceived that self-management support reduces the cost of care for people with CLBP and the health system by enhancing the efficiency of care (e.g., reducing strain on the health system). However, it is essential to highlight that there were some notable differences in the understanding of the aims, processes, and outcomes of self-management support between physiotherapists and medical doctors. For instance, physiotherapist participants emphasized the use of exercise as a strategy to help patients gain function. In contrast, medical doctors explained the goal of self-management as a means to reduce the burden on the health system without consideration of the patient’s functional gains. Such differences in the beliefs and understanding of self-management support could be attributed to the disciplinary orientation of the participants. Regardless, the self-management support benefits discussed by the participants has significant implications for Ethiopia. First, the majority of the Ethiopian population have to travel a long distance to access care (non-direct medical cost) and make out-of-pocket payments to access health care services (direct medical cost) [62, 63], hence saving costs related to managing pain for people with CLBP. Second, self-management support benefits the health system by reducing demand on the already overstretched health system with limited resources.

Participants in this study viewed patient-centeredness as imperative to the facilitation of self-management support for people with CLBP. The concepts identified in this study: *patients need to be active participants, and patients bring their own self-management approach* are consistent with the notion that people with a chronic health condition, not the health care providers, are at the center of managing their condition during self-management support process [26, 64–66]. This finding underscores the providers’ understanding of patients’ roles in their self-management process. Our results align with findings from research on self-management of people with chronic musculoskeletal pain conditions, including LBP. Their motivation, active participation, and commitment to engage in their self-management process were significant predictors of self-management outcomes [20, 67]. Furthermore, our results highlight the importance of valuing patient’s self-management strategies and engaging them in the decision-making process about their self-management options.

In this study, the participants depicted a number of barriers presenting challenges to provide self-management support for people with CLBP. These barriers were related to health care providers’ lack of competency to provide self-management support, health system factors, and patient factors. Although the HCPs acknowledged their role in providing self-management support for people with CLBP to self-manage, there is a lack of perceived competency to design and implement patient-tailored self-management support for people with the condition. It is also important to note that the participants emphasized on the need for specific and generic competencies related to self-management support of people with CLBP, such as conducting proper assessment and diagnosis, communicating patients in a simple language appropriate to the patient, and teamwork and cooperation with other members of the health care team) [59, 68–70].

The health system-related barriers described by the participants in this study, such as – patient overload, poor referral system, and lack of strategies to objectively measure outcome of self-management support- are universal [71, 72]. Health systems worldwide, including that of Ethiopia, are oriented towards acute health problems in a biomedical model where appointments are brief and episodic [73–75]. Evidence shows that self-management is less prioritized and patients are less likely engaged in a decision-making process in a biomedically oriented and busy medical encounter [71, 72]. However, it is important to note that a misperception by HCPs that patients have to travel to health care settings, rather than community settings, for self-management support may underlie some of the health systems barriers (e.g., patient overload and lack of time) discussed by a few participants.

Lastly, consistent with previous reports, patient-related potential obstacles to attending self-management in this study included a low level of engagement in the self-management process, an education level (e.g., low literacy level) [76], and residence (e.g., living in the rural area) [77]. In Ethiopia, people from rural areas are often less educated, do not have access to transportation and the financial capability to travel to cities to access health care services or attend self-management support. Patient-related factors such as preferences for medical management (e.g., injection) coupled with a negative outlook towards the self-management approach were the attitudinal barriers to providing non-pharmacological self-management support for people with CLBP reported in this study [78].

Our findings suggest that a few HCPs used pain education to reassure their patients that there is nothing critically wrong with their low back and it is okay to engage in activities of daily living. This is in line with previous studies suggesting the importance of pain reassurance as an important strategy to facilitate self-management support for people with LBP [79, 80]. Additionally, the providers in this study recommended or used a wide range of self-management support strategies, including active approaches (e.g., exercises and activity modifications) [59, 81–83], passive approaches (e.g., modalities and medications) [33, 82, 84], patient education, and family engagement [34, 85, 86] to help patients self-manage. Although these self-management support strategies are consistent with previous findings, most of these strategies may not necessarily be based on self-management support principles [87]. The main focus of self-management support is enhancing patient’s skills and confidence to self-manage the day-to-day health challenges (e.g., pain and its impacts) through guidance from their HCPs [20, 31, 88]. These skills include problem-solving, decision making, resource utilization, the formation of a patient-provider partnership, goal setting, action planning, and self-tailoring [26, 31], which were all missing from the strategies that providers identified. As reflected throughout this study, the providers had both literal (e.g., meaning of the term self-management in Amharic) and conceptual misunderstanding of self-management support for people with CLBP.

The lack of proper understanding of self-management support is a good indicator that healthcare providers do not receive education on self-management as part of their training [89, 90]. Such a gap may become an obstacle to designing and delivering evidence-based self-management support for people with CLBP in Ethiopia. As Ethiopia faces an increasing burden of chronic diseases [91, 92], training competent health care professionals who can successfully support people with chronic conditions to self-manage should be a priority. The development and implementation of CLBP self-management in Ethiopia is unimaginable without competent health care providers who can deliver it. As such, Ethiopia’s education policy should accommodate and reflect the challenges chronic conditions are putting on the health system. Preparing the health care workforce and equipping them with competencies to manage chronic conditions, including CLBP, is paramount. The inclusion of self-management content in entry-level health care providers’ curricula should be among the priorities.

This study is not without limitations. First, the study only provided an insight into the health care providers’ understanding of self-management and self-management support. The inclusion of patients’ views in this study would have provided a deeper understanding of the concept and meaning of CLBP self-management in Ethiopia. We recommend future studies to investigate patient’s views on this concept to understand if their perspectives are consistent with the health care providers. Second, we did not conduct inter-coder reliability during the coding process. Instead, we used a reflective approach to understand whether there is consistency in the data coding process and conceptual similarity and difference in the codes between the two coders. Lastly, member checking was not done to confirm the validity of the themes. The final stage of data analysis was conducted in Canada and getting feedback on the themes from the participants who reside in Ethiopia was not possible due to logistical challenges.

Overall, the findings from this study provide foundational knowledge to design and implement context-specific CLBP self-management support in Ethiopia. As indicated by the providers, several contextual factors can facilitate or hinder patients’ self-management process in the Ethiopian context. Therefore, the design and implementation of CLBP self-management support must consider patients’ socio-demographic variables such as literacy level, residence, income, culture, and religious practices.

## Conclusion

In conclusion, although HCPs in this study are open and appreciated the potential of self-management support for people with CLBP, the concept is new to many of them. There also exist several barriers to facilitate self-management support for people with CLBP in Ethiopia. One of these barriers was related to health care providers’ lack of perceived competency to provide self-management support. Many of the support strategies suggested by the HCPs mainly focused on improving impairments rather than helping patients build self-efficacy skills. This finding underlines the need for comprehensive training to enhance self-management support competencies for Ethiopian HCPs. Lastly, this study’s findings have clinical and research implications for informing the design and implementation of future context-based CLBP self-management support in Ethiopia. Future research could also focus on improving the existing health system to support the design and integration of self-management support in the Ethiopian context.

## Supplementary Information

**Additional file 1.**

## Data Availability

Data for this study is available and can be provided by the corresponding author upon request.
