# Supplementary material for: Health care providers’ understanding of self-management support for people with chronic low back pain in Ethiopia: an interpretive description
Source: BMC Health Serv Res. 2022 Feb 14;22:194. doi: 10.1186/s12913-022-07610-5 (PMC8842538; doi:10.1186/s12913-022-07610-5)
Supplement: Supplementary file 1 — Additional file 1. [file 12913_2022_7610_MOESM1_ESM.docx]

**Appendix**

**Themes, sub-themes, and quotes for study II**

| **Themes related to self-management and self-management support** | **Participants’ quotes** |
| --- | --- |
| Self-management is a new concept | “Self-management may have various meanings when used in different sentences. But when we translate it directly [from English to Amharic], it may mean self-treatment or  self-care.” [HCP 009; Physiotherapist] |
| Self-management has potential benefits | “The patient won’t make frequent visits every time they have pain- they manage it themselves when their pain comes back. They won’t be absent from work too.” [HCP 002; Physiotherapist] |
| Patient-centeredness is key to facilitating self-management support | “It is self-management! Patients manage their pain by themselves based on the advice they get from medical professionals. Controlling their pain is ultimately their role. Isn’t that what the phrase suggests? It is a hundred percent their role. In a hospital setting, the patient’s roles are limited. However, if it is self-management, patients take the role of managing their pain.” [HCP 007; Neurologist] |
| There are barriers to providing self-management support | “There is also a limitation from the physician’s side. Hmm, we use all our experiences and read books on this concept [self-management]. But applying that in practice on a patient is our limitation. So, lack of knowledge is the first thing. And then how to apply self-management is the next.” [HCP 012; Orthopedic surgeon]  “As I said, … history taking, conducting a physical examination and giving patients advice and so on require lots of time. You do not have the luxury of time to allocate that much time and bond with them [patients]. That is a challenge.” [HCP 006; Orthopedic surgeon] |
| Participants have diverse views on self-management support | “Women use a very big ‘*meqenet*’ [traditional belt made of clothes] to comfort their back. To me, it is like a brace to support their back. They may need to use it mostly when they work, sometimes even when they sleep.” [HCP 008; Orthopedic surgeon]  “At a chronic stage, patients have already adapted to their pain, so what they need from you is more reassurance. So, you tell them that they are fine and continue whatever self-management intervention is given to them.” [HCP 012; Orthopedic surgeon]  “No- it has to be patient-centered. You have to always take the patient’s history, quality of life, income level, and other factors into consideration. Otherwise, you can’t just design management for them before you consider those things.” [HCP 001; Physiotherapist] |
| Specific competencies are required to facilitate self-management support | “You must be able to diagnose the cause of the pain...different causes of back pain may need different self-management...so it requires a competency to be able to say what the cause of the pain is.” [HCP 006; Orthopedic surgeon] |
| There are lots of opportunities to design self-management support for people with CLBP | “Other than that, I see most patients come with family or friends. They don’t come alone. I think it is good- they [family and friends] can remind the patients to follow the advice we give them.” [HCP 012; Orthopedic surgeon]  “Other than that, I think it is possible to engage the patient’s close family members to support them in the process.” [HCP 023; Physiotherapist]  “Now there are good things- we have an increasing number of specialist and subspecialist doctors in the country.” [HCP 018; Neurologist]  “I see a flare-up in the number of physicians. There is an increasing number of graduating physicians. I see it as an advantage to train more people with this skill in the country.” [HCP 016; Neurosurgeon].  “…the educated ones, those who can read and write can at least go on the internet, find some resources, download and use it when you tell them about self-management” [HCP 021; Physiotherapist].  “You do not need lots of equipment to manage low back pain. What you need is a floor and maybe one wall. Material is not a limitation. Patients can use their body weight to exercise.” [HCP 019; Physiotherapist] |

**Sample interview guide for health care providers**

**Study title**: exploring the understanding, perspectives, and experiences of healthcare providers in Ethiopia on self-management for adults with chronic low back pain

1. What comes to your mind when you hear the phrase self-management?

**Probing questions**: what is self-management from your perspective?

1. How do you define the term “Self-management?”
2. When you think about chronic low back pain self-management interventions, what are your perceptions of them?

**Probing questions:**

- What makes self-management interventions different from other forms of interventions for people with chronic low back pain?
- what is the ultimate goal of chronic low back pain self-management interventions?
- In what ways do you think is self-management is important for people with chronic low back pain?
- What roles should patients with CLBP play to self-manage their condition?

1. What is your role in helping people with chronic low back pain self-manage their condition?

**Probing questions:**

- How well do you think you currently help people with chronic low back pain to self-manage their condition? Is teaching self-management part of your usual involvement with a patient?
- What strategies do you use to support patients with chronic low back pain self-manage their condition?
- How do you do that?

1. What knowledge and skills do healthcare professionals need to facilitate chronic low back pain self-management?

**Probing questions**: what competencies do healthcare professionals need to deliver/ facilitate CLBP SM intervention?

1. Can you tell me the most exciting and challenging part of facilitating self-management for people with chronic low back pain in your institution?
2. Is there anything related to chronic low back pain self-management that we haven’t discussed today that you would like to share with us?
